# Severity of COVID-19–Related Illness in Massachusetts, July 2021 to December 2022

**DOI:** 10.1001/jamanetworkopen.2023.8203

**Published:** 2023-04-13

**Authors:** Alaleh Azhir, Zachary H. Strasser, Shawn N. Murphy, Hossein Estiri

**Affiliations:** 1 Harvard-MIT (Massachusetts Institute of Technology) Program in Health Sciences and Technology, Harvard Medical School, Boston, Massachusetts; 2 Clinical Augmented Intelligence Group, Laboratory of Computer Science, Department of Medicine, Massachusetts General Hospital, Boston, Massachusetts; 3 Department of Medicine, Harvard Medical School, Boston, Massachusetts; 4 Department of Neurology, Massachusetts General Hospital, Boston, Massachusetts

## Abstract

This cohort study uses hospitalization and 30-day mortality risks to create a temporal profile of the severity of COVID-19 in Massachusetts from July 2021 to December 2022.

## Introduction

Recent SARS-CoV-2 variants have been associated with lower severity.^1,2,3^ Estimating the true severity of SARS-CoV-2 across time is difficult due to its rapid mutations,^4,5^ changing immunity profiles (eg, vaccinations and prior infections), and health care system capabilities (eg, new therapeutics). In this retrospective cohort study, while accounting for a comprehensive set of confounders including demographic characteristics, immunity, therapeutics, and comorbidities, we constructed a temporal profile of SARS-CoV-2’s severity in Massachusetts, measured through hospitalization and 30-day mortality risks, and hypothesized that severity was diminishing.

## Methods

We constructed an incident-level database of SARS-CoV-2 cases recorded within Mass General Brigham between July 1, 2021, and December 31, 2022. The use of data was approved by the Mass General Brigham institutional review board, with a waiver of informed consent for the use of deidentified data. Our study followed the STROBE reporting guideline.

We performed a weighted causal inference study using methods described by Strasser et al.^6^ Cases were categorized based on the infection month, with November 2021 as the comparison benchmark. Covariates including the patient demographic characteristics, therapeutics, comorbidity score, vaccination status, and previous SARS-CoV-2 infection were adjusted using entropy balancing to reduce confounding bias. Outcomes were hospitalization and in-hospital death within 30 days of a positive SARS-CoV-2 test result. We computed weighted odds ratios and 95% CIs between November 2021 and other months with a survey-weighted logistic regression model. Further, we stratified our cases by vaccination status (unvaccinated vs ≥2 vaccines) and calculated similar treatment outcomes between unvaccinated individuals in November 2021 and vaccinated and unvaccinated individuals in all other months. Last, we calculated the adjusted absolute risk difference and ratio of hospitalization and mortality between the vaccinated and unvaccinated groups (eMethods in Supplement 1).

## Results

Of 195 150 cases (mean [SD] age, 48 [22] years; 62% women and 38% men), 19% were unvaccinated (Table). By self-reported race, 4% were Asian; 6%, Black; 81%, White; and 9%, other (including American Indian or Alaska Native, Arab, Native Hawaiian or other Pacific Islander, multiple races, or other) or unknown. After covariate adjustment, the weighted odds of hospitalization and 30-day mortality decreased over months, overall and by vaccination status (Figure). Being vaccinated was associated with decreased odds of hospitalization in all months and of mortality in all months except April, November, and December 2022. Overall, vaccinated compared with unvaccinated individuals had 40 (95% CI, 35-44) fewer hospitalizations and 6 (95% CI, 4-7) fewer deaths per 1000 cases. For an average person, the risk of hospitalization was 55% (95% CI, 52%-58%) lower and mortality was 68% (95% CI, 60%-73%) lower had they been vaccinated, not accounting for time from vaccination.

**Table.** Descriptive Statistics of Study Cohort per Month

Characterisic	Study year and month^a^																	
	2021						2022											
	Jul	Aug	Sep	Oct	Nov	Dec	Jan	Feb	Mar	Apr	May	Jun	Jul	Aug	Sep	Oct	Nov	Dec
No. of cases	1140	3760	3820	3520	5720	22 400	35 710	4830	4180	12 200	23 890	13 250	13 170	5140	11 760	10 890	7780	11 980
Sex																		
Men	482 (42)	1541 (41)	1574 (41)	1480 (42)	2338 (41)	8707 (39)	13 392 (38)	1846 (38)	1571 (38)	4428 (36)	8684 (36)	4960 (37)	4880 (37)	1886 (37)	4388 (37)	3979 (37)	2934 (38)	4412 (37)
Women	658 (58)	2219 (59)	2246 (59)	2040 (58)	3382 (59)	13 693 (61)	22 318 (63)	2984 (62)	2609 (62)	7772 (64)	15 206 (64)	8290 (63)	8290 (63)	3245 (63)	7372 (63)	6911 (64)	4846 (62)	7568 (63)
Age, mean (SD)	43 (21)	43 (22)	44 (22)	46 (23)	43 (22)	41 (21)	42 (21)	46 (22)	46 (21)	48 (20)	50 (20)	50 (20)	51 (20)	51 (20)	54 (20)	57 (20)	56 (20)	56 (20)
Race																		
Asian	42 (4)	89 (2)	85 (2)	90 (3)	166 (3)	696 (3)	1574 (4)	220 (5)	225 (5)	591 (5)	1297 (5)	795 (6)	720 (5)	280 (5)	527 (4)	481 (4)	311 (4)	433 (4)
Black	120 (11)	385 (10)	293 (8)	184 (5)	317 (6)	1887 (8)	3263 (9)	265 (5)	174 (4)	496 (4)	985 (4)	591 (4)	674 (5)	307 (6)	533 (5)	462 (4)	382 (5)	602 (5)
White	879 (77)	2850 (76)	3060 (80)	2940 (84)	4658 (81)	16 862 (75)	25 519 (71)	3881 (80)	3489 (83)	10 318 (85)	19 776 (83)	10 842 (82)	10 682 (81)	4120 (80)	9794 (83)	9213 (85)	6403 (82)	9872 (82)
Other or unknown^b^	113 (10)	427 (11)	426 (11)	325 (9)	588 (10)	2975 (13)	5358 (15)	438 (9)	283 (7)	895 (7)	1852 (8)	1046 (8)	1122 (9)	451 (9)	904 (8)	733 (7)	674 (9)	1074 (9)
Unvaccinated	548 (487)	1786 (48)	1770 (46)	1473 (42)	2506 (44)	6906 (31)	8831 (25)	1014 (21)	513 (12)	1243 (10)	2368 (10)	1267 (10)	1238 (9)	560 (11)	1198 (10)	1015 (9)	777 (10)	1132 (9)
≥2 Vaccines	561 (49)	1866 (50)	1951 (51)	1923 (55)	2971 (52)	14 547 (65)	25 395 (71)	3570 (74)	3526 (84)	10 645 (87)	20 784 (87)	11 564 (87)	11 557 (88)	4449 (87)	10 213 (87)	9533 (88)	6762 (87)	10 454 (87)
Prior infection	44 (4)	75 (2)	58 (2)	60 (2)	84 (1)	1049 (5)	2262 (6)	299 (6)	169 (4)	537 (4)	1249 (5)	914 (7)	1131 (9)	560 (11)	1178 (10)	1114 (10)	1079 (14)	2588 (22)
Corticosteroid treatment	75 (7)	245 (7)	258 (7)	243 (7)	348 (6)	900 (4)	1281 (4)	288 (6)	210 (5)	432 (4)	783 (3)	453 (3)	528 (4)	204 (4)	494 (4)	556 (5)	398 (5)	704 (6)
Paxlovid	0	0	0	0	0	1 (0.004)	28 (0.08)	95 (2)	511 (12)	3041 (25)	6582 (28)	3738 (28)	4018 (31)	1564 (30)	3503 (30)	3095 (28)	2282 (29)	3977 (33)
Remedsivir	45 (4)	144 (4)	162 (4)	133 (4)	212 (4)	506 (2)	713 (2)	134 (3)	86 (2)	212 (2)	370 (2)	215 (2)	275 (2)	139 (3)	258 (2)	335 (3)	355 (5)	684 (6)
Elixhauser comorbidity index, mean (SD)	1.54 (2.3)	1.52 (2.3)	1.63 (2.3)	1.74 (2.4)	1.67 (2.3)	1.53 (2.2)	1.72 (2.4)	2.04 (2.8)	1.85 (2.5)	1.93 (2.4)	1.98 (2.4)	2.07 (2.5)	2.32 (2.6)	2.46 (2.7)	2.50 (2.7)	2.67 (2.8)	2.77 (3.0)	2.93 (3.0)
Hospitalization	87 (8)	209 (6)	223 (6)	195 (6)	269 (5)	661 (3)	929 (3)	339 (7)	180 (4)	323 (3)	590 (2)	358 (3)	479 (4)	218 (4)	442 (4)	536 (5)	413 (5)	808 (7)
Mortality	8 (0.70)	16 (0.43)	23 (0.60)	26 (0.73)	51 (0.89)	78 (0.35)	132 (0.37)	30 (0.62)	25 (0.60)	27 (0.22)	48 (0.20)	24 (0.18)	30 (0.23)	17 (0.33)	44 (0.37)	48 (0.44)	43 (0.55)	54 (0.45)

^a^ Unless otherwise indicated, data are expressed as No. (%) of cases. Numbers are obfuscated (thus sums may not add up to true marginal totals), and percentages are rounded.

^b^ Other includes American Indian or Alaska Native, Arab, Native Hawaiian or other Pacific Islander, multiple races, or other. Unknown refers to unknown or declined to answer. Race was self-identified by patients within their electronic health record.

**Figure.**
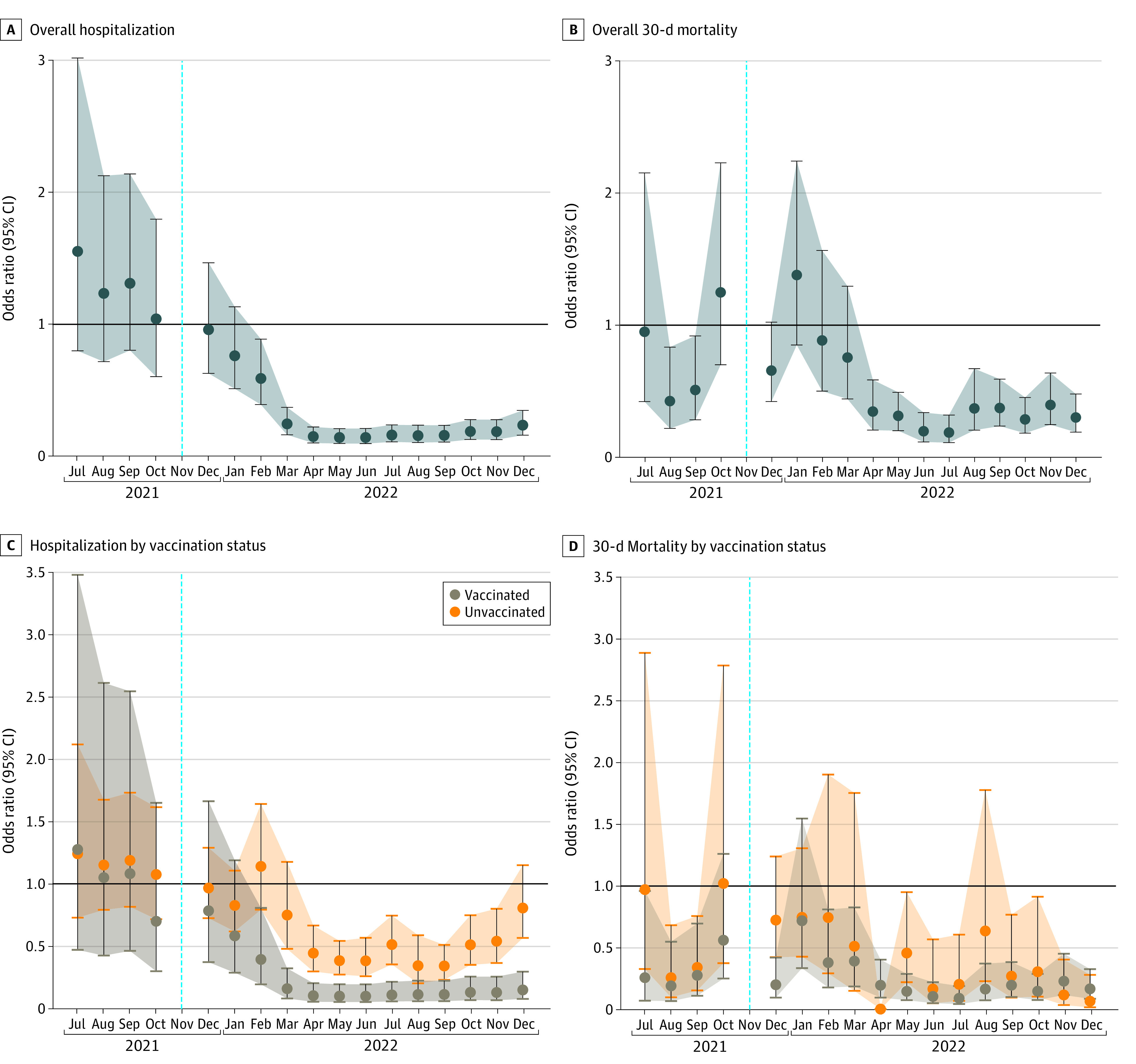
Study Cohort Outcome Risks Hospitalization (A) and 30-day mortality risk (B) of cases detected in each month compared with that of November 2021. C and D, Hospitalization (C) and 30-day mortality risk (D) of vaccinated and unvaccinated cases detected in each month compared with unvaccinated cases in November 2021. Dotted blue vertical lines indicate the benchmark date of November 2021; whiskers and shaded areas indicate 95% CIs.

## Discussion

This cohort study shows that SARS-CoV-2’s severity waned between July 2021 and December 2022 after adjusting for confounders regardless of vaccination status. Overall, vaccination is still associated with a decrease in risk of severe SARS-CoV-2 outcomes. Use of entropy balancing provides an advantage over standard matching, as unmatched individuals are not discarded, which increases effect estimates’ precision and findings’ generalizability. With the SARS-CoV-2 variants demonstrating milder severity over recent months, it is reasonable to presume that our findings are broadly generalizable. Our models can be easily deployed across varying locations to assess temporal severity of SARS-CoV-2.

Study limitations include vaccinated and unvaccinated populations not being balanced when examining severity over time, not accounting for time from vaccination, unobserved SARS-CoV-2 infections not detected by a positive test result or electronic health record flag, and unobserved vaccination at another facility. Mortality is underestimated by only including in-hospital deaths within 30 days; however, this is evenly distributed across months. Other unmeasured confounders may remain.

## Notes

Supplement 1. **eMethods.** Cohort, Definitions, Sample Size, and Data Analysis

Supplement 2.
Data Sharing Statement

## References

[1] Nealon J, Cowling BJ. Omicron severity: milder but not mild. Lancet. 2022;399(10323):412-413. doi:10.1016/S0140-6736(22)00056-3 35065007 PMC8769661

[2] Wolter N, Jassat W, Walaza S, . Early assessment of the clinical severity of the SARS-CoV-2 omicron variant in South Africa: a data linkage study. Lancet. 2022;399(10323):437-446. doi:10.1016/S0140-6736(22)00017-4 35065011 PMC8769664

[3] Ulloa AC, Buchan SA, Daneman N, Brown KA. Estimates of SARS-CoV-2 Omicron variant severity in Ontario, Canada. JAMA. 2022;327(13):1286-1288. doi:10.1001/jama.2022.2274 35175280 PMC8855311

[4] Fan Y, Li X, Zhang L, Wan S, Zhang L, Zhou F. SARS-CoV-2 Omicron variant: recent progress and future perspectives. Signal Transduct Target Ther. 2022;7(1):141. doi:10.1038/s41392-022-00997-x 35484110 PMC9047469

[5] Kimura I, Yamasoba D, Tamura T, ; Genotype to Phenotype Japan (G2P-Japan) Consortium. Virological characteristics of the SARS-CoV-2 Omicron BA.2 subvariants, including BA.4 and BA.5. Cell. 2022;185(21):3992-4007.e16. doi:10.1016/j.cell.2022.09.018 36198317 PMC9472642

[6] Strasser ZH, Greifer N, Hadavand A, Murphy SN, Estiri H. Estimates of SARS-CoV-2 Omicron BA.2 subvariant severity in New England. JAMA Netw Open. 2022;5(10):e2238354. doi:10.1001/jamanetworkopen.2022.38354 36282501 PMC9597387

