# Supplementary material for: Severity of COVID-19–Related Illness in Massachusetts, July 2021 to December 2022
Source: JAMA Netw Open. 2023 Apr 13;6(4):e238203. doi: 10.1001/jamanetworkopen.2023.8203 (PMC10102873; doi:10.1001/jamanetworkopen.2023.8203)
Supplement: Supplement 1. — eMethods. Cohort, Definitions, Sample Size, and Data Analysis [file jamanetwopen-e238203-s001.pdf]

Supplementary Online Content

Azhir A, Strasser ZH, Murphy SN, Estiri H. Severity of COVID-19–related illness in Massachusetts, July 2021 to December 2022. *JAMA Netw Open*. 2023;6(4):e238203. doi:10.1001/jamanetworkopen.2023.8203

eMethods. Cohort, Definitions, Sample Size, and Data Analysis

This supplementary material has been provided by the authors to give readers additional information about their work.

© 2023 Azhir A et al. *JAMA Network Open*.

eMethods. Cohort, Definitions, Sample Size, and Data Analysis

Cohort Assembly

Study was approved by the Mass General Brigham (MGB) institutional review board protocol # 2020P001063. Patients were identified as SARS-CoV-2 positive, by having either a positive polymerase chain reaction (PCR) result either inside or outside of the MGB system within Epic's EHR, or having a positive at-home antigen test and having that be recorded by a provider. To minimize missingness in comorbidity history and follow-up care, we applied a minimum data floor threshold of at least two diagnosis records, six months apart, in the three years prior to the SARS-CoV-2 infection.

Incidence Definition and covariates

The first positive PCR for a patient was considered the incident date of their disease. If a patient had another positive PCR or new flag greater than 90 days from the incident date, this was considered to be a new case of COVID-19.

We integrated electronic health records obtained through Covid-19 Data Mart (which includes Research Patient Data Registry and Enterprise Data Warehouse) and state-level vaccine registry data obtained through Covid-19 Vaccine Registry for each SARS-CoV-2 infection incidence. We then constructed variables describing the patient's age, Elixhauser comorbidity index score, vaccination status, race, sex, prior COVID infection, and use of antiviral therapy (nirmatrelvir/ritonavir or remdesivir) or steroids (dexamethasone). Race was self-identified by patients within their electronic health record. Elixhauster comorbidity score was calculated by entering International Classification of Diseases 10 codes into R package "comorbidity".

Sample Size Calculation

Due to the descriptive nature of this study, sample sizes were not computed, and the cohort size was fixed per chosen criteria. Adequacy of the sample size was assessed by the confidence intervals around the primary point estimates.

© 2023 Azhir A et al. *JAMA Network Open*.

Statistical Analysis

To construct the temporal severity profile, we categorized cases in 18 groups based on the infection month (July 2021 to December 2022), with November 2021 as the comparison benchmark. Using a weighted causal inference approach, we applied entropy balancing for weighing the covariates with the average treatment effect (ATE) estimand, where month of infection was the treatment variable. We then computed weighted odds ratios and confidence intervals between the month of November 2021 and all other months with a survey-weighted logistic regression model.

All statistical analysis was performed in R version 4.1.2. The R package ‘Weightit’ was used for estimating the entropy balancing weights, the R package ‘cobalt’ was used for checking balance, and the R package ‘survey’ was used to estimate the weighted odds ratios and confidence intervals. The code for analysis is available at: https://github.com/Alaleh1191/Covid-19/

MGB COVID-19 vaccine registry

The MGB COVID-19 vaccine registry includes patient vaccination data (dose number, brand, and date) for patients with a record of receiving any type or dose of COVID-19 vaccine. The registry combined administrations recorded from 3 workflows: (1) MGB COVID vaccination administration, (2) Epic documentation by clinician based on patient’s report of COVID vaccination, and (3) a record from MIIS (Massachusetts Immunization Information System, which may be reconciled or not yet reconciled by clinicians in EPIC). The MIIS record is updated weekly but may be an underestimation of the number of patients who are getting vaccination elsewhere due to query limits per day.

Data Sharing Statement

The data was extracted from Mass General Brigham's COVID-19 Data Mart Enclave and Covid-19 Vaccine Registry. Due to privacy regulations and per institutional and IRB approvals for this study, the patient level data cannot be shared.

© 2023 Azhir A et al. *JAMA Network Open*.
