# Supplementary material for: Severity of COVID-19–Related Illness in Massachusetts, July 2021 to December 2022
Source: JAMA Netw Open. 2023 Apr 13;6(4):e238203. doi: 10.1001/jamanetworkopen.2023.8203 (PMC10102873; doi:10.1001/jamanetworkopen.2023.8203)
Supplement: Supplement 2. — Data Sharing Statement [file jamanetwopen-e238203-s002.pdf]

Data Sharing Statement

Azhir. Severity of COVID-19-Related Illness in Massachusetts, July 2021 to December 2022. *JAMA Netw Open*. Published April 13, 2023. doi:10.1001/jamanetworkopen.2023.8203

Data
Data available: No

Additional Information
Explanation for why data not available: The data was extracted from Mass General Brigham's COVID-19 Data Mart Enclave and Covid-19 Vaccine Registry. Due to privacy regulations and per institutional and IRB approvals for this study, the patient level data cannot be shared.
